# Exploring Forsterite Surface Catalysis in HCN Polymerization: Computational Insights for Astrobiology and Prebiotic Chemistry

**DOI:** 10.1021/acsearthspacechem.4c00282

**Published:** 2025-01-17

**Authors:** Niccolò Bancone, Stefano Pantaleone, Piero Ugliengo, Albert Rimola, Marta Corno

**Affiliations:** † Departament de Química, Universitat Autònoma de Barcelona, Bellaterra, Catalonia 08193, Spain; ‡ Dipartimento di Chimica and Nanostructured Interfaces and Surfaces (NIS) Centre, Università degli Studi di Torino, via P. Giuria 7, Torino 10125, Italy

**Keywords:** IR, ISM, astrochemistry, DFT, kinetics, silicates, comets, meteorites, adenine

## Abstract

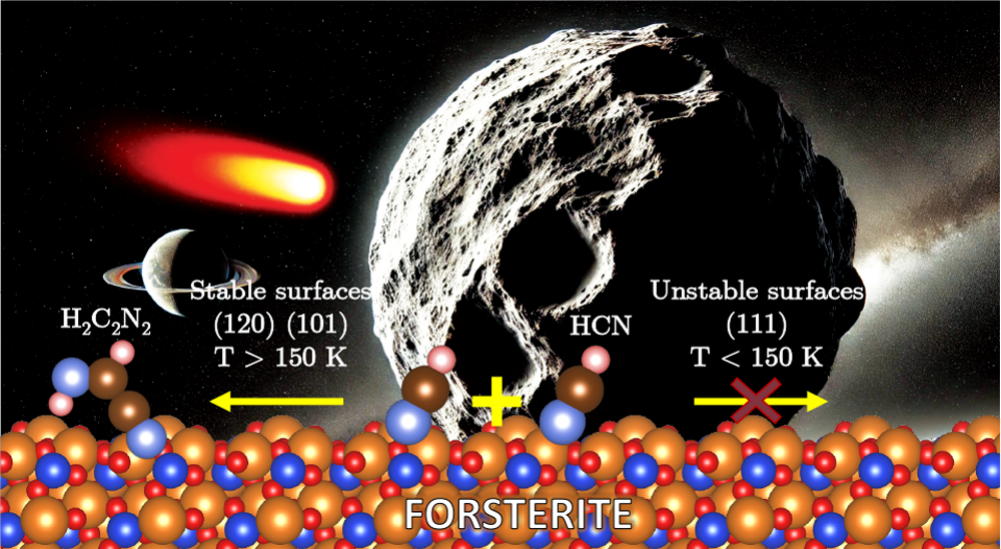

Understanding the catalytic role of cosmic mineral surfaces is crucial for elucidating the chemical evolution needed for the emergence of life on Earth and other planetary systems. In this study, the catalytic role of silicate forsterite (Mg_2_SiO_4_) surfaces in the synthesis of iminoacetonitrile (IAN, HN=CH–CN) from the condensation of two hydrogen cyanide (HCN) molecules is investigated through quantum mechanical simulations. Using density functional theory calculations, the potential energy surfaces alongside the kinetics of various surface-mediated reactions leading to the formation of IAN are characterized. The effectiveness of forsterite as a catalyst is a delicate balance of the surface reactivity: on one side, the deprotonation of HCN is mandatory to trigger the dimerization; on the other side, the species should be weakly bound to the surface, thus allowing for their diffusion to meet with each other. The work reveals interesting counterintuitive results: the (120) and (101) forsterite surfaces (the less reactive ones) exhibit favorable catalytic properties for the reaction, in detriment to the (111) one (one of the most reactive). The implications of these findings in the astrobiology and prebiotic chemistry fields and for laboratory experiments are discussed, highlighting the potential role of cosmic silicates in the synthesis of complex organic molecules.

## Introduction

Hydrogen cyanide (HCN) is a molecule of paramount importance in the origin of life studies^1,2^ since 1961, when its polymerization carried out by J. Oró to synthesize adenine gave rise to one of the milestones of prebiotic chemistry.^3^

To date, HCN molecules are well-known to be widespread in the interstellar medium (ISM): they have been detected in different environments, including molecular clouds,^4,5^ protostellar hot cores,^6−8^ and other astrophysical bodies and habitats, such as carbonaceous chondrite meteorites,^9^ cometary comas,^10−12^ and planetary atmospheres.^13−17^ Similarly, possible products of HCN polymerization have been observed in astrophysical environments typical of the latest stages of a planetary system formation, such as comets,^18^ local planets, and satellites,^19−21^ while more generically purines have been detected in the organic fraction of the Murchison Meteorite and Ryugu asteroid.^22,23^ In this regard, HCN molecules could have been building blocks of more complex species of prebiotic interest in these environments, like adenine, which results from the polymerization of five HCN molecules. Moreover, the presence of prebiotic molecules on “carrier” bodies like meteorites and comets supports the possibility of external inputs of biomolecules on newly forming planets when a comet passes close to or a meteorite strikes them.

Accordingly, a considerable effort has been made in the last decades to shed light on the mechanisms of polymerization of HCN under interstellar and protoplanetary conditions.^24−26^ The polymerization of HCN to adenine is postulated to start with nucleophilic attacks by the C-end of CN^–^ to the neutral H_*x*_C_*x*_N_*x*_ backbone.^3^ The first step is thus the dimerization of HCN, giving rise to iminoacetonitrile (IAN, HN=CH–CN). However, the gas-phase dimerization of neutral HCN leads to another isomer of IAN (namely, methanimidoyl isocyanide, HN=CH–NC), which involves the formation of a N–C covalent bond instead of C–C. At this point, a C≡N–N≡C– isomerization would be necessary to form a C–C covalent bond, but the HN=CH– *cis–trans* isomerization is more favored instead.^25^ When the reaction involves hydrogen isocyanide (HNC, the isomer of HCN), the dimerization reaction can lead to the formation of either a C–N or C–C bond, the latter channel (the one that ultimately leads to adenine) being more favored. However, even in this case, the reaction presents high energy barriers, still hampering efficient kinetics at the ISM conditions.^27^ The introduction of basic or acidic catalysts facilitates the reactivity of HCN in the gas-phase,^25,26,28^ suggesting that, even in the inert conditions of the ISM, at least the first steps of the HCN oligomerization can take place.

Despite the difficulties encountered in the gas-phase, both experimental and theoretical results evidence that HCN can successfully polymerize in the polar liquid-phase, where acid–base mechanisms are more prone to occur.^3,29−31^ More generally, this suggests that the polymerization of HCN might take place in condensed phases (either liquid or solid state) with the presence of catalysts. In this scenario, a fundamental role is played by the surfaces of cosmic grains composing the rocky fraction of planets, satellites, meteorites, and cometary nuclei. One of the main constituents of the grains is silicates (ubiquitous in several astrophysical environments), which are the forge of molecular hydrogen and the first simple molecules in the ISM, synthesized by the hydrogenation of atomic species that freeze on the grain from the gas phase, and which in turn form the icy mantle covering the rocky core (H_2_O, CH_3_OH, CH_4_, and NH_3_).^32−39^ This opens the question on whether or not grain surfaces could also play a role in the chemistry of HCN, catalyzing the formation of HCN polymers up to nucleobases. On this topic, the catalytic role of water ice mantles toward the dimerization of HCN has already been investigated theoretically, showing a sensible reduction of the activation barrier (from ∼300 to ∼150–170 kJ mol^–1^).^40^

When a protostar forms in the center of a molecular cloud, the increase of temperature in the protostellar envelope causes the sublimation of the icy mantle of the grains,^41^ allowing a clean exposition of the catalytic surfaces of the rocky core of the grains to the environmental gas. Additionally, the increasing temperature with respect to molecular clouds enables the catalytic role of such surfaces in the polymerization of HCN. This feature has been recently investigated after initiation by siloxyl radicals, showing that at the ISM conditions, HCN can easily oligomerize to 1,3,5-triazine or be hydrogenated to form amino-methanol.^42^ An experimental work investigated the adsorption of pure HCN and its polymerization on different mineral surfaces, including forsterite (Mg_2_SiO_4_, the pure Mg end-member of olivines).^43^ A considerable reactivity above 300 K was reported, with adenine formation among the other products, suggesting that HCN can successfully polymerize at the proto-planetary, meteoritic, and/or cometary conditions.

Nevertheless, the atomistic details of the HCN polymerization on silicate surfaces are still unknown, and accordingly, in this work, we modeled the dimerization of HCN adsorbed on different surfaces of crystalline Mg_2_SiO_4_ by means of a density functional theory (DFT) approach. We focus on this material as olivines tend to be more abundant than pyroxenes in carbonaceous chondrites,^44^ and the content of Mg in cosmic silicates tends to be higher than Fe, likely due to the higher solubility of Fe silicates.^45^ Thus, such crystalline models aim to mimic natural Mg_2_SiO_4_ surfaces present in asteroids, cometary nuclei, and planetary environments, where crystalline silicate fractions are dominant with respect to the amorphous ones, which, on the contrary, represent the major phase in pristine dust grains in interstellar molecular clouds.^46,47^

Starting with a previous study of ours on the adsorption of HCN on six forsterite surfaces, i.e., (010), (120), (101), (001), (111), and (021),^48,49^ we selected three of them with different stabilities ((120), (101), and (111)) and modeled a total amount of six HCN dimerization reactive channels to form IAN as the reaction product, adopting a Langmuir–Hinshelwood mechanism.

## Methods

All calculations were carried out with the CRYSTAL17 code,^50^ which implements wave function and DFT methods for periodic systems by applying translational operators to localized Gaussian-type orbitals (GTOs). Geometry optimizations and frequency calculations were carried out at the PBE DFT level of theory,^51^ corrected for the Grimme’s revised D*N empirical term to include dispersion interactions.^52−54^ Well aware of the importance of choosing an adequate DFT method for describing reacting organic systems,^55^ we performed a preliminary benchmarking study to assess the performance of different functionals in computing energy barriers and reaction energies for the HCN dimerization to IAN on a (Mg_2_SiO_4_)_3_ forsterite nanocluster.^56−58^ DFT results computed with CRYSTAL17 were compared with those at the DLPNO-CCSD(T)/aug-cc-pvtz level of theory computed with the ORCA code, v5.0.3.^59^ (see Table S1 and Figure S1 for details).

The BHLYP functional,^60,61^ corrected for the Grimme’s D3 empirical scheme with the Becke–Johnson (BJ) damping,^62−64^ provided the best agreement with DLPNO-CCSD(T), with absolute percentage deviations of 3.8 and 16% on the potential energy barrier and reaction energy, respectively. For this reason, single-point energy calculations at the BHLYP-D3(BJ) theory level on the PBE-D*N-optimized geometries (BHLYP-D3(BJ)//PBE-D*N) were carried out to improve the accuracy of the energetics of the reaction when simulated on the periodic silicate surfaces.

In accordance with our previous works on the HCN adsorption on forsterite surfaces,^48,49^ we adopted the same GTOs proposed by Bruno et al.^65^ as the basis set for describing forsterite atoms, and the richer Ahlrichs VTZP basis set^66^ augmented with polarization functions on H, C, and N. Tolerances of the integral calculation were set to 10^–6^ for Coulomb overlap, Coulomb penetration, exchange overlap, and exchange pseudo-overlap in the direct space, and 10^–14^ for exchange pseudo-overlap in the reciprocal space. The electron charge density was numerically integrated by adopting an extralarge grid, and the reciprocal lattice was sampled by a grid of 5 *k*-points in the first Brillouin zone. Geometry optimizations were performed by adopting the Broyden–Fletcher–Goldfarb–Shanno (BFGS) algorithm.^67−70^ The threshold values on the SCF energy were set to 10^–6^, 10^–7^, 10^–10^, and 10^–11^*E*_h_ for single-point, minima optimizations, frequency calculations, and transition states optimizations, respectively. Transition states (TSs) were localized by adopting the distinguished reaction coordinate (DRC)^71,72^ approach implemented in CRYSTAL17.

The vibrational frequencies of each stationary point were computed numerically through the central difference formula; that is, the second derivatives of the potential energy surface (PES) were calculated by manually displacing each atom from its equilibrium position along each Cartesian coordinate by ±0.003 Å. In order to speed up such calculations, only those atoms belonging to the adsorbates and to the first layer of the surfaces were allowed to be displaced. By proceeding so, we could confirm the real nature of minima points of the PES, for which no imaginary frequencies are expected, and of the saddle points (TS), which are in turn characterized by one and only one imaginary frequency associated with the reaction coordinate.

All the potential energy values (*E*) were corrected for the zero-point energy (ZPE), allowing us to compute the relative enthalpies at 0 K (Δ*H*(0 K)) as
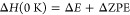
1

Thermal corrections were added to Δ*H* when computing the relative Gibbs energies as
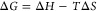
2

In order to derive the kinetic constants for the reactions, we applied the RRKM theory,^73−76^ which was adapted for surface reactions and implemented in a homemade code freely available.^77^ See Supporting Information for more details on the RRKM scheme.

Based on their relative stability, the HCN dimerization reaction was simulated on the Mg_2_SiO_4_ (120), (101), and (111) periodic slab models, as representative surfaces of forsterite (of high, intermediate, and low stability, respectively). They belong to the groups of faces constituting the forsterite crystal morphology, and have been well characterized in previous theoretical works.^48,65,78^ Details of these surfaces can be found in the Supporting Information.

## Results and Discussion

### HCN Dimerization in the Gas Phase

**Figure 1:**
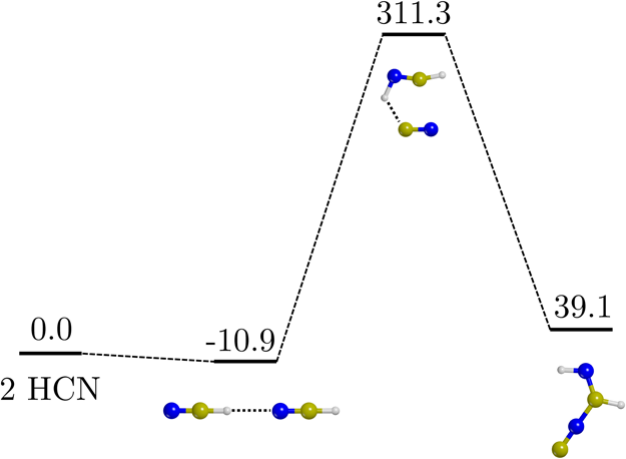
ZPE-corrected PES (Δ*H*(0 K)) of the HCN dimerization in the gas phase calculated at the BHLYP-D3(BJ)//PBE-D*N level of theory. Values are in kJ mol^–1^. Color code: N in blue, C in ochre, and H in white.

Figure 1 shows the PES of the HCN dimerization in the gas phase, adopting the mechanism proposed in ref (25), and computed at the BHLYP-D3(BJ)//PBE-D*N theory level.

In agreement with the current literature,^24^ the main obstacles to this pathway are its high energy barrier (311.3 kJ mol^–1^) and its endothermicity (39.1 kJ mol^–1^), which prevent the reaction from occurring at any appreciable rate at the interstellar conditions. Moreover, as mentioned in the Introduction, this non-catalyzed reaction inevitably leads to the methanimidoyl isocyanide isomer. The only way to obtain the actual IAN in the gas phase is by using HNC as a reactive species, whose reaction, in any case, also presents very high activation barriers (∼300 kJ mol^–1^).^40^

The formation of a C–C bond is a well-established task in organic synthesis; a fundamental aspect is the formation of a nucleophilic carbon that attacks an electrophilic one. To this end, the easiest way to obtain a nucleophile/electrophile pair is by deprotonating one of the two HCN molecules with a base (B), in such a way that the HCN dimerization reaction occurs through the following elementary steps:


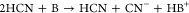



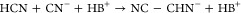



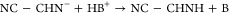


The first step is HCN deprotonation by B generating a CN^–^ anion as the nucleophile, while the unperturbed HCN becomes the electrophile. The second step is the nucleophilic attack of the CN^–^ to HCN, forming a C–C bond and NC–CHN^–^, the deprotonated IAN (IAN^–^). Finally, IAN^–^ is protonated by the conjugated acid (HB^+^) of the starting base. Depending on the nature of B, the above-mentioned steps can also be barrier-less or concerted, as we will show in the following.

In addition to creating a nucleophile/electrophile pair, making the nucleophilic attack of CN^–^ efficient is also an interesting point to enhance the rate of the reaction. This can be achieved by increasing the electrophilicity of HCN, for example, by coordinating the N-end terminus to a positive or partially positive acidic species, like the H atom of a third HCN,^30^ the H atom of a water molecule,^29,40^ or the conjugated acid of a base,^29^ the effect of which is a dramatic decrease of the activation barrier to ∼91–92 kJ mol^–1^.^29,30^

Forsterite surfaces, thanks to the interplay between the exposed Lewis acidic Mg^2+^ and Lewis basic O^2–^, allow the above-mentioned points: the adsorption of HCN on the most unstable surfaces induces the deprotonation of HCN,^48,49^ and the interaction of a nondeprotonated HCN molecule with the stable surface increases its electrophilic character. These two features are key ingredients toward the synthesis of IAN (C–C bond), as it is shown in the following section.

### HCN Dimerization on Forsterite Surfaces

As previously mentioned, in this study, six different Mg_2_SiO_4_ surfaces have been used as catalysts for the HCN dimerization to form the IAN adopting a Langmuir–Hinshelwood mechanism.

In previous works on forsterite surface modeling,^65,78^ the relative stability of the six different surfaces constituting mainly its crystal morphology, based on their surface energy (*E*_S_), is (from more to less stable): (010) > (120) > (101) > (001) > (111) > (021). Since the relative stability of a surface is an indicator of its reactivity and contribution to the surface area (i.e., the more stable, the more extended, and the less reactive the surface, and vice versa), we chose three representative facets, namely, the (120), (101) and (111) ones, presenting high, intermediate, and low stabilities, respectively.

**Figure 2:**
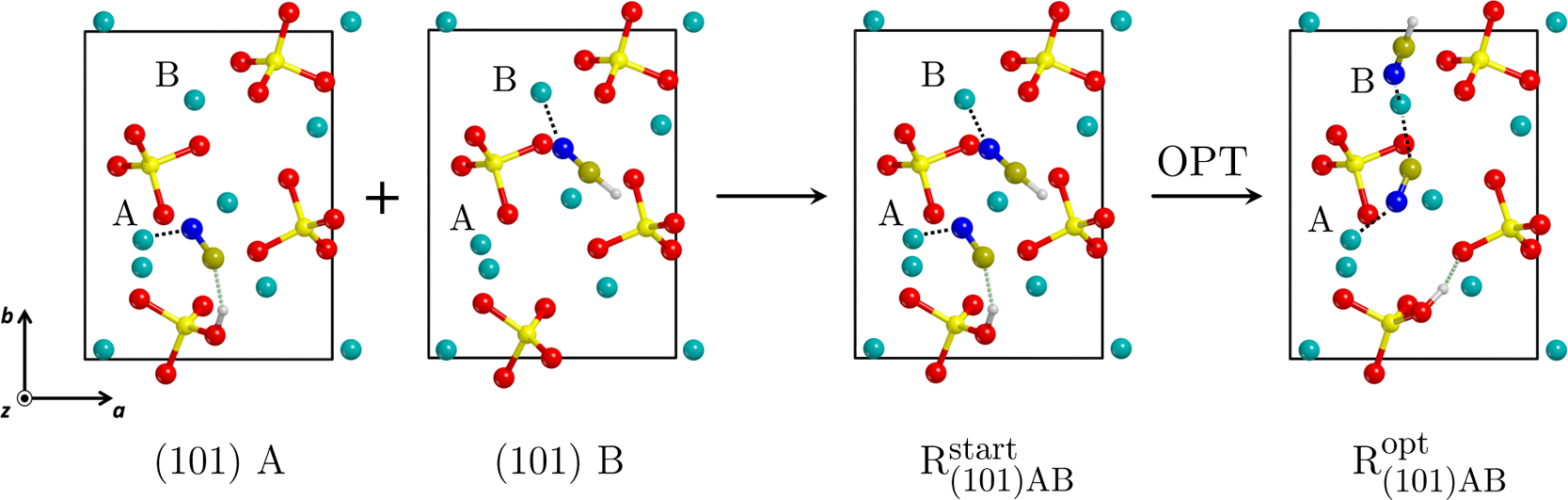
Scheme of the modeling strategy used in this work to obtain the initial reactant structures, using the (101) surface as a representative case: (i) two HCN molecules are placed on the surface at the corresponding geometries obtained in ref (48), and (ii) the geometry is optimized at the PBE-D*N level . Color code: Mg in cyan, Si in yellow, O in red, N in blue, C in ochre, and H in white.

Figure 2 shows the procedure to obtain the initial guess structure for the reactants. We retrieved the starting structures from our previous papers in which single HCN···forsterite adsorption complexes were studied (i.e., one HCN molecule per unit cell). Since the surfaces expose several Mg^2+^ adsorption sites, many complexes were explored for each surface. Here, we generated the reactants by first placing two HCN molecules on the surface, at the same geometry previously obtained for the respective single adsorptions, optimizing afterward the new guess structure with the double HCN adsorption. Please note that the same nomenclature of the binding sites as adopted in previous works^48,79^ is used (see Figure S2 for details), and accordingly the resulting reactant complexes are named as R_(120)BC_, R_(101)AB_, R_(101)BC_, R_(111)BC_, R_(111)BD_, and R_(111)DE_.

As mentioned above, forsterite surfaces expose both Lewis basic O and Lewis acidic Mg species.^80^ The former can efficiently deprotonate HCN, resulting in the adsorption of a CN^–^ anion and a surface silanol (Si–OH) group (see, for instance, structure (101) A in Figure 2). Indeed, in several cases relative to HCN single adsorption, the deprotonation of the HCN molecules was spontaneously observed upon adsorption (i.e., during the optimization of the structure), particularly on reactive (namely, less stable) surfaces, like the (101) and (111) ones. In contrast, on the (120) surface (the most stable out of the three), no spontaneous deprotonation of HCN was observed; however, an exoergonic deprotonation (Δ*G*^dept^ = −7.6 and −6.0 kJ mol^–1^ at 10 and 300 K, respectively) and a small Gibbs energy barrier (Δ*G*^‡,dept^ = 2.5 and 9.7 kJ mol^–1^ at 10 and 300 K, respectively) were found,^48^ indicating the feasibility of the HCN deprotonation also on this surface at these conditions.

Table 1 reports the energetics (i.e., Gibbs energy barriers and reaction Gibbs energies at 10, 50, 150, 200, and 300 K), kinetic rate constants, and half-life times at these temperatures for all the reactions.

**Table 1:** Gibbs Energy Barriers and Reaction Gibbs Energies (Δ*G*^‡^ and Δ*G*, Respectively, in kJ mol^–1^), Unimolecular Kinetic Rate Constants (*k*, in s^–1^), and Associated Half Lifetimes (*t*_1/2_, in h)^a^

		temperature (K)		
case		150	200	300
(120)BC	Δ*G*^‡,dept^	25.0	25.9	28.2
	Δ*G*^dept^	–51.1	–50.7	–49.7
	*k*^dept^	1.4 × 10^5^	5.9 × 10^6^	3.6 × 10^8^
		1.4 × 10^–9^	3.3 × 10^–11^	5.3 × 10^–13^
	Δ*G*^‡,CC^	90.4	91.9	95.8
	Δ*G*^CC^	26.6	28.0	31.5
	*k*^CC^	3.5 × 10^–19^	1.3 × 10^–11^	4.3 × 10^–4^
		5.5 × 10^14^	1.5 × 10^7^	0.45
(101)AB	Δ*G*^‡,CC^	74.7	75.0	76.1
	Δ*G*^CC^	65.7	66.1	67.3
	*k*^CC^	3.7 × 10^–14^	7.8 × 10^–8^	0.13
		5.2 × 10^9^	2.5 × 10^3^	1.4 × 10^–3^
(101) BC	Δ*G*^‡,CC^	52.6	52.8	53.6
	Δ*G*^CC^	–53.9	–52.5	–48.8
	*k*^CC^	1.8 × 10^–6^	8.1 × 10^–02^	3.4 × 10^3^
		1.1 × 10^2^	2.4 × 10^–3^	5.7 × 10^–8^
(111)BC	Δ*G*^‡,CC^	174.3	175.1	177.5
	Δ*G*^CC^	156.1	156.5	157.9
	*k*^CC^	0	0	7.9 × 10^–17^
		*∞*	*∞*	2.5 × 10^12^
(111)BD	Δ*G*^‡,CC^	123.9	124.8	126.8
	Δ*G*^CC^	94.4	95.5	98.1
	*k*^CC^	0	0	4.3 × 10^–10^
		*∞*	*∞*	4.5 × 10^5^
(111)DE	Δ*G*^‡,CC^	143.0	143.4	144.5
	Δ*G*^CC^	144.8	145.1	146.1
	*k*^CC^	0	0	6.7 × 10^–11^
		*∞*	*∞*	2.9 × 10^6^

a *k* values smaller than 10^–19^ s^–1^ are treated as zero. The superscript “dept” refers to the deprotonation of HCN, and the superscript “CC” refers to the C–C bond formation.

**Figure 3:**
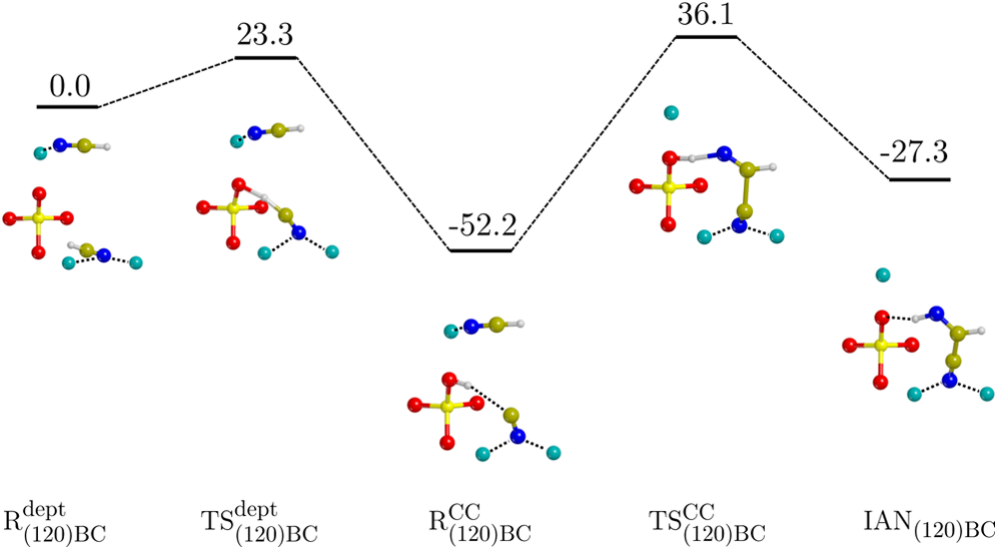
ZPE-corrected PES (Δ*H*(0 K)) for the HCN dimerization on the (120) surface calculated at the BHLYP-D3(BJ)//PBE-D*N level of theory. The superscript “dept” refers to the deprotonation of HCN, and the superscript “CC” refers to the C–C bond formation. Values are in kJ mol^–1^. Color code: Mg in cyan, Si in yellow, O in red, N in blue, C in ochre, and H in white.

The ZPE-corrected PES for the (120)BC case is shown in Figure 3. According to what is mentioned above, the deprotonation of HCN on the (120) surface is exothermic (Δ*H*^dept^(0 K) = −52.2 kJ mol^–1^) with the associated barrier relatively low (Δ*H*^dept^(0 K) = 23.3 kJ mol^–1^). The subsequent C–C bond formation proceeds with the migration of the HCN toward the CN^–^. This diffusion breaks the Mg^2+^···N dative bond and the Si–OH···CN hydrogen bond (H-bond), and a proton transfer from the silanol to the product occurs concertedly, meaning that, in this case, the step gives rise directly to IAN without passing through the IAN^–^ anionic form as a preproduct. Although the elementary step associated with the IAN_(120)BC_ formation is endothermic (Δ*H*^CC^(0 K) = 24.9 kJ mol^–1^ with respect to the  intermediate), the overall reaction energy is exothermic (Δ*H*^CC^(0 K) = −27.3 kJ mol^–1^). The reaction presents two energy barriers, the highest one being that associated with the C–C bond formation, (Δ*H*^‡,CC^(0 K) = 88.3 kJ mol^–1^ with respect to the  intermediate structure, namely, the intrinsic energy barrier).

**Figure 4:**
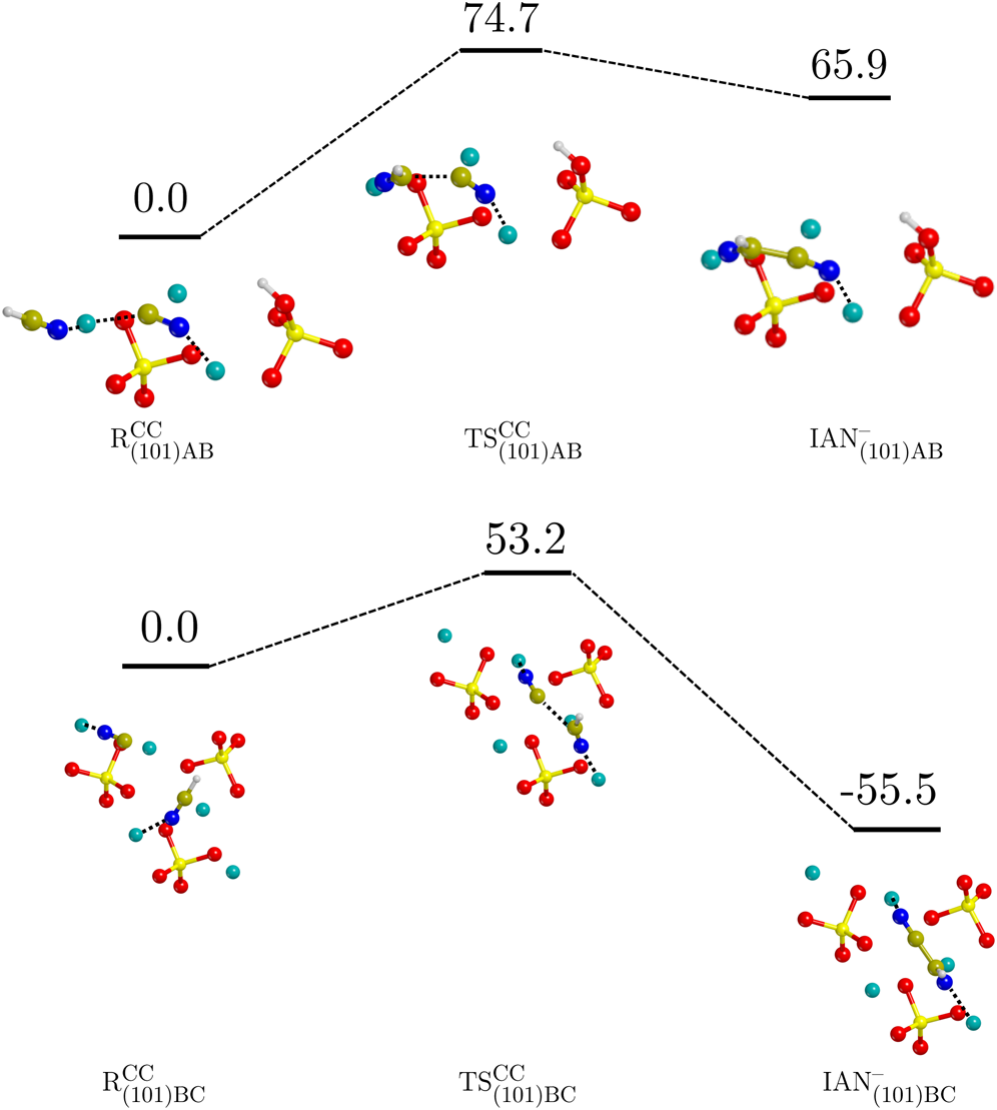
ZPE-corrected PES (Δ*H*(0 K)) for the HCN dimerization on the (101) surface calculated at the BHLYP-D3(BJ)//PBE-D*N level of theory. The superscript “CC” refers to the C–C bond formation. Values are in kJ mol^–1^. Color code: Mg in cyan, Si in yellow, O in red, N in blue, C in ochre, and H in white.

Figure 4 shows the ZPE-corrected PESs for the two cases occurring on the (101) surface, namely, (101)AB and (101)BC. Here, at variance with the (120)BC case, the reactant structures present an already deprotonated CN^–^ molecule, while the other is HCN. We assume this configuration because, as mentioned above, spontaneous deprotonation of HCN upon adsorption occurs on the involved surface binding sites.^48^ As the initial reactant structures present the nucleophilic CN^–^, the identified reactive mechanisms present a single step, which is C–C bond formation. For both simulated reactions, the energy barriers are smaller (Δ*H*^‡,CC^(0 K) = 74.7 and 53.2 kJ mol^–1^ for the (101)AB and (101)BC cases, respectively) than that on the (120) surface (Δ*H*^‡,CC^(0 K) = 88.3 kJ/mol). Energy barriers reported in the literature for the HCN dimerization in the liquid phase are 91.6 kJ mol^–1^ for the ammonia-catalyzed reaction in implicit water solvent (at the MP2/6-31++G** level of theory),^29^ 91.2 kJ mol^–1^ for the self-catalyzed reaction in liquid HCN at 278 K (PBE-D3 refined with B3LYP-D3),^30^ and 141 kJ mol^–1^ for the dimerization on water ice surfaces in implicit water solvent (B3LYP/aug-cc-pVQZ).^40^ Accordingly, it turns out that the barriers predicted in this work are, to the best of our knowledge, the lowest ones identified so far, pointing out significant catalytic activity of the forsterite surfaces.

The calculated reaction energies are deeply different between the (101)AB and (101)BC cases (Δ*H*^CC^(0 K) = 65.9 and −55.5 kJ mol^–1^, respectively). The discrepancy can be ascribed to the different interaction strength of the reactive species with the binding sites (R_(101)AB_ being more favorable than R_(101)BC_, see Table 2), which define the 0 energy reference of respective PESs. It is thus clear that the same reactants and products on different binding sites do not present the same binding energy (Table 2). That is, if we sum-up the difference in binding energy (BE) between the products and the reactants we obtain , which is close to  (please note that the binding energies are calculated at PBE-D*N//PBE-D*N level of theory from ref (48), while reaction energies at BHLYP-D3(BJ)//PBE-D*N). Accordingly, this does not necessarily mean that the formation of IAN_(101)AB_^–^ is disfavored with respect to IAN_(101)BC_^–^, but only that the difference in the thermodynamics of the two sites can be simply explained with the adsorption energies: in the specific, large part of the difference in  is due to the reactants, which are clearly placed on different wells of the PES. As a further confirmation the BEs of IAN_(101)AB_^–^ and IAN_(101)BC_^–^ are similar, indicating that the relative stability of the two products is very similar. One possibility is that IAN_(101)AB_^–^ back-reacts to , the backward barrier being only Δ*H*^‡,CC^(0 K) = 8.8 kJ mol^–1^, and therefore  is less effective than  as a catalytic site.

**Table 2:** PBE-D*N//PBE-D*N Adsorption Energies of All of the Considered Reactions^a^

	case					
	(120)BC	(101)AB	(101)BC	(111)BC	(111)BD	(111)DE
R^dept^	–163.7					
R^CC^	–192.9	–426.8	–348.4	–426.7	–383.4	–364.6
IAN^a^/IAN^–b^	–160.2^a^	–333.0^b^	–364.7^b^	–238.8^b^	–276.2^b^	–222.0^b^

a The superscript “dept” refers to the deprotonation of HCN, and the superscript “CC” refers to the C–C bond formation. The superscripts “a” and “b” refer to the molecular and deprotonated IAN, respectively. Values are in kJ mol^–1^.

**Figure 5:**
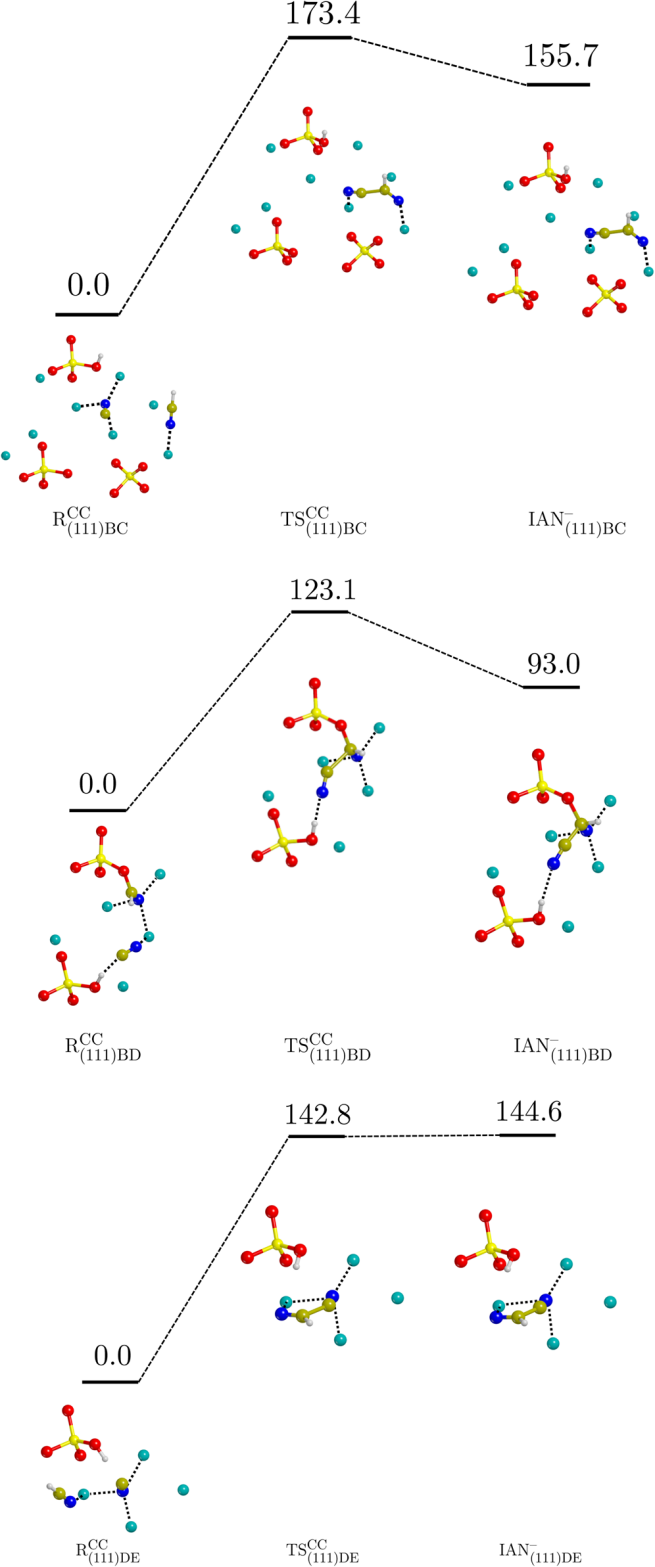
ZPE-corrected PES (Δ*H*(0 K)) for the HCN dimerization on the (111) surface calculated at the BHLYP-D3(BJ)//PBE-D*N level of theory. The superscript “CC” refers to the C–C bond formation. Values are in kJ mol^–1^. Color code: Mg in cyan, Si in yellow, O in red, N in blue, C in ochre, and H in white.

Figure 5 reports the ZPE-corrected PESs of the three reactions explored on the (111) surface, namely, the (111)BC, (111)BD, and (111)DE cases. As for the (101) cases, the reactants involve already deprotonated CN^–^ for the same reasons. The three reactions show higher energy barriers than the previous cases, with Δ*H*^‡,CC^(0 K) spanning from 123.1 to 173.4 kJ mol^–1^. For what concerns the (111)DE case, the reaction enthalpy at 0 K results to be higher than the barrier, as the former is 144.6 and the latter 142.8 kJ mol^–1^. This inversion of stability between the TS and the product occurs after the ZPE corrections are applied to the potential energies computed at the BHLYP-D3(BJ) theory level. Indeed, the potential energy barrier and reaction energy before including the ZPE corrections are 142.1 and 140.7 kJ mol^–1^, respectively. The energies computed at the PBE-D*N level follow the same trend, being the energy barrier and the reaction energy, respectively, 81.2 and 79.0 kJ mol^–1^ without the ZPE corrections, and 81.9 and 82.9 kJ mol^–1^ including ZPE corrections. Thus, the inversion of stability is observed at both the PBE-D*N and BHLYP-D3(BJ) levels of theory. Therefore, (111)DE could be either barrier-less or present an energy difference between TS and product which lays within the DFT accuracy (∼4 kJ mol^–1^). Nevertheless, the reaction is thermodynamically disfavored and does not represent a significant HCN dimerization channel in astronomical environments.

The same comparison between ΔΔ*H*^CC^(0 K) and ΔBE as for the (101) surface can be applied to the cases on the (111) facet. However, it is more interesting to extend the comparison among different surfaces than on different sites of the same surface. Indeed, as shown in Table 2, the reactants on the binding sites of the (101) and (111) facets present relatively similar BEs, while the thermodynamics of the reactions are very different, particularly on the (111), where all the reactions are strongly disfavored. The reason is related to the adsorption energies of the IAN product on the (111) surface, which are ∼100–200 kJ mol^–1^ higher (i.e., less stable) than those on the (101) surface. The direct consequence of the lower stability of the product on the (111) binding sites is a significant increase in energy barrier, hindering the dimerization of HCN even at high temperatures.

**Figure 6:**
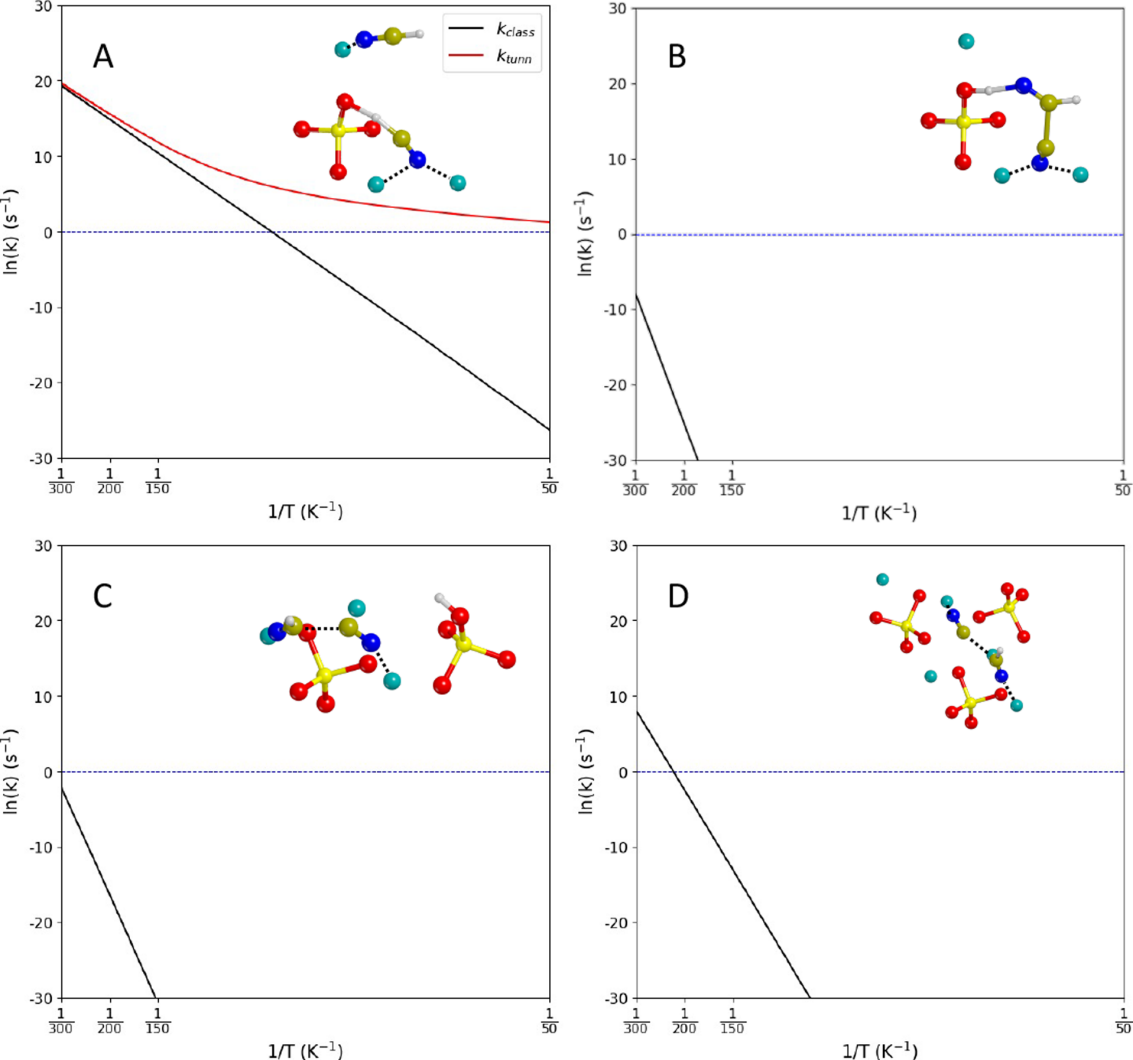
Arrhenius plots between 50 and 300 K for the deprotonation of HCN in the (120)BC case (TS_(120)BC_^dept^, panel A), and the C–C bond formation in the (120)BC case (TS_(120)BC_^CC^, panel B), the (101)AB case (TS_(101)AB_^CC^, panel C), and the (101)BC case (TS_(101)BC_^CC^, panel D). Black lines: plots computed classically (*k*_class_). Red line: plot including Eckart tunneling (*k*_tunn_).

Overall, thermal corrections do not change the thermodynamics of the reactions; in contrast, the kinetic rate constants are strongly affected by the temperature, spanning many orders of magnitude between 150 and 300 K. Therefore, for the three cases with the lowest energy barriers (namely, the (120)BC, (101)AB, and (101)BC cases), the Arrhenius plots between 50 and 300 K are represented in Figure 6. As regards the deprotonation of HCN for the (120)BC case (Figure 6A), quantum tunneling effects clearly dominate the process at low temperatures (consistent with the particle-like nature of the process, i.e., a proton transfer), resulting in kinetic rate constants higher than 1 s^–1^ between 50 and 300 K. As regards the C–C formation, if we take the  structure as the 0-energy reference, the reaction presents a relatively low barrier (36.6 kJ mol^–1^), which can easily be overcome if the nascent energy released by the deprotonation (i.e., Δ*H*^dept^(0K) – Δ*H*^‡,dept^(0K) = −75.5 kJ mol^–1^) is channeled into the reaction path and not dispersed by the coupling with lattice phonons.^37−39^ However, in the case that the reaction energy dissipation is fast (i.e., less than a picosecond), it is then more physically sound to take  as the intrinsic 0-energy reference for the actual C–C bond formation step. In this scenario, the time scale of the reaction is appreciably fast at high temperatures, with *t*_1/2_ at 300 K of 0.45 h.

The calculated reaction rates involving the (101)AB case indicate that the reaction is feasible (considering astronomical time scales) only within the 200–300 K range, due to its relatively high Gibbs energy barrier (Δ*G*^‡,CC^(200 K) = 75.0 kJ mol^–1^), while that involving the (101)BC case is appreciably faster at 150 K (Δ*G*^‡,CC^(150 K) = 52.6 kJ mol^–1^).

### Comparison with Laboratory Experiments and Astronomical Observations

Laboratory experiments confirm that HCN molecules start to successfully oligomerize on forsterite surfaces at 300 K, its tetramer diaminomaleonitrile being the major product; at low temperatures (i.e., 150 K), physisorption is mainly observed, even if new bands with very small intensities rise, indicating that a small quantity of HCN reacts.^43,49^ To date, there is no experimental detection of IAN in reacting mixtures of HCN, even at low temperatures, as it is commonly considered as a short-lived intermediate in the prebiotic oligomerization of HCN.

Based on the values reported in Table 1, we show that three cases, i.e., (120)BC, (101)AB, and (101)BC, present reaction rates that are in line with the experimental findings. At 300 K, on the (101) surface, the IAN formation reactions are almost instantaneous (*t*_1/2_ of the order of 10^–3^ and 10^–7^ h), and on the (120) surface, *t*_1/2_ is of few seconds. At 150 K, the comparison with the experiments is reasonable, as the (101)BC case presents reaction rates compatible with laboratory time scales, indicating that small quantities of HCN can react even at low temperatures. Additionally, according to the thermodynamics, the formation of IAN is endothermic (Δ*G*_(120)BC_^CC^ (300 K) = 31.5, Δ*G*_(101)AB_^CC^ (300 K) = 67.3 kJ mol^–1^) or slightly exothermic (Δ*G*_(101)BC_^CC^ (300 K) = −48.8 kJ mol^–1^), meaning that IAN is a transient intermediate in the HCN polymerization chain that rapidly evolves toward the formation of the stable diaminomaleonitrile. Accordingly, these results indicate that isolating IAN as a reaction intermediate in the presence of high HCN loadings is difficult, which is also in agreement with the experimental measurements.

**Figure 7:**
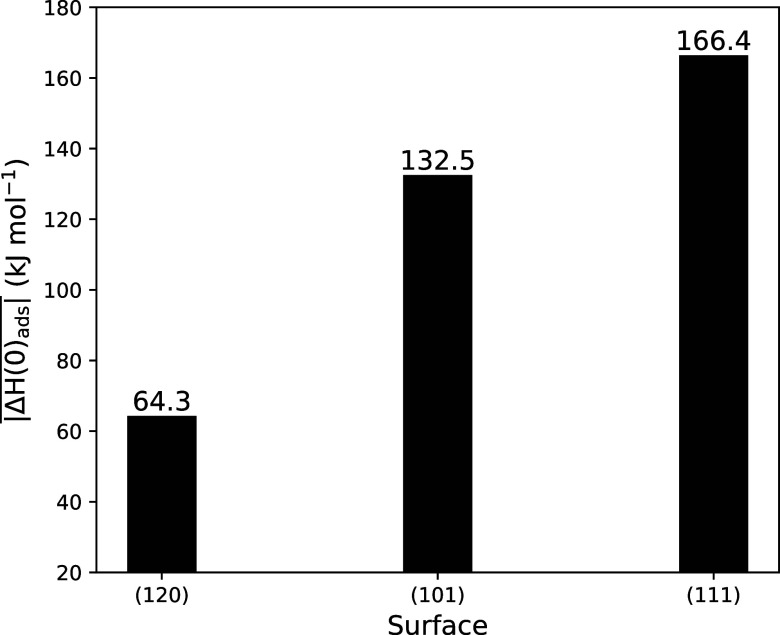
Average adsorption enthalpies at 0 K (in absolute values) as a result of the different adsorption complexes of HCN on the forsterite (120), (101), and (111) surfaces, computed at the PBE-D*N//PBE-D*N level. Data from ref (48) .

In contrast to the capability of the (120) and (101) surfaces to catalyze the HCN dimerization, all the studied cases on the (111) surface suffer from both unfavored thermodynamics and kinetics in the explored temperature range. This trend is consistent with the higher stability of the (120) and (101) surfaces compared to the (111) surface, as well as with the corresponding binding energies of HCN on these surfaces.^48^Figure 7 represents the average adsorption enthalpies at 0 K (in absolute values) on the (120), (101), and (111) surfaces from ref (48). It is clear that the less stable the surface, the stronger the binding of HCN. We have also shown that unstable surfaces expose more Lewis basic O^2–^ anions, which are more prone to deprotonate HCN upon adsorption, forming CN^–^. On the other hand, if the surface is unstable, potential reactive HCN and CN^–^ adsorbed on the surface are bound too strongly, hindering them to react to form the C–C bond. Thus, the effectiveness of forsterite in catalyzing the reaction is a delicate trade-off between these two phenomena. This is actually true for the (120) and (101) surfaces, in which adsorbed HCN and CN^–^ coexist and their binding energies allow them to react to form IAN. However, on the (111), although HCN and CN^–^ are available on the surface, the binding is so strong that it keeps them stuck without any possibility to react. Remarkably, since the largest contributions to the surface area of crystalline nanoparticles are due to the most stable surfaces, we can conclude that forsterite, as a whole, is very active toward HCN polymerization.

In addition, our prediction indicates that, in the absence of photoprocessing, cosmic rays, and/or stellar winds, the reaction occurs thanks to Lewis acid/base catalysis, which in any case requires relatively high temperatures. This could be consistent with the possible production of HCN-polymers and HCN-derived prebiotic molecules in warmer and more evolved astrophysical environments and bodies, such as comets, meteorites, and proto-planetary atmospheres,^18−22^ indeed supporting the crucial role of temperature for the reaction to take place. Water could further help the process, facilitating proton transfers and stabilizing the TS of the reactions, for which further studies are required.

Finally, we conclude with a short consideration about the structural state (namely, crystalline vs amorphous phases) of forsterite. Even if theoretical studies point out that crystalline and amorphous silicates, unlike silica, may not be easily distinguished by IR emission observations,^81,82^ amorphous olivines are supposed to be dominant in the composition of the rocky core of interstellar dust grains (around 95%,^47^), while the crystalline phase increases during the accretion of the grains in a planetary system formation.^46^Figure S3 reports the surface energies of all the studied forsterite facets as calculated in our previous work.^48^ We remind the reader that the values are potential energies calculated at the PBE-D*N//PBE-D*N level. However, the relative energies and the morphological relevant indexes (MRI) are very similar among previous works using different levels of theory.^48,65,79^ One can see that the (120) and (101) surfaces show high MRI values (33.0 and 21.8%, respectively), meaning that they tend to be more extended than other surfaces such as the (111) one (MRI = 3.3%). This further supports the catalytic effectiveness of natural forsterite crystals, as they averagely expose more stable and catalytically active surfaces.

Amorphous Mg_2_SiO_4_ surfaces usually expose more basic anions with respect to the crystalline phase. This is not because amorphous silicates are more basic than crystalline ones, but due to the presence of MgO_*x*_ islands in the material.^80,82^ A higher number of basic sites implies a higher degree of HCN deprotonation, and accordingly, a higher number of dimerization reactions can be triggered on amorphous Mg_2_SiO_4_ surfaces. Indeed, it has been experimentally demonstrated that amorphous Mg_2_SiO_4_ possesses an enhanced catalytic activity over the crystalline one toward the HCN prebiotic polymerization,^43^ which is reflected by the large production and detection of heavier products than the tetramer diaminomaleonitrile.

## Conclusions

In this study, DFT calculations were used to explore the energetics and the kinetics of the prebiotic dimerization of HCN to iminoacetonitrile on different facets of forsterite surfaces through a Langmuir–Hinshelwood mechanism.

In accordance with the mechanisms currently accepted for HCN dimerization in polar condensed phases, the presence of Lewis basic O^2–^ sites on the forsterite surfaces facilitates the deprotonation of adsorbed HCN molecules, leading to the formation of cyanide ions (CN^–^), which are powerful nucleophiles in the dimerization reaction. The transition states associated with the condensation of CN^–^ and HCN species are moreover efficiently stabilized by superficial Lewis acidic Mg^2+^ sites or exposed silanols of the surfaces.

Our results indicate that the effectiveness of forsterite in catalyzing the HCN dimerization process is strongly influenced by the specific surface orientation. According to calculated thermodynamic and kinetic data, the reaction is more feasible on the more stable (120) and (101) surfaces rather than the less stable (111) surface, meaning that the majority of the available surface area of forsterite nanoparticles presents active sites for HCN polymerization.

On the basis of our results, we suggest caution when adopting the definition “unstable surface ≡ reactive surface”, as, in some cases like those discussed here, the catalytic effectiveness of the material lays in a compromise between its ability of activating the reactants and their binding on the surface, which in the case to be strong prevents the progress of the reaction.

Fast kinetics occurs at the temperature conditions proper of evolved astronomical bodies like comets, meteorites, and planetary atmospheres, in accordance with observations of HCN-derived products in such environments (150–300 K), as well as at the temperatures where laboratory experiments have indeed observed HCN polymerization (≥300 K).

Our findings provide insights into the catalytic mechanisms of forsterite surfaces in prebiotic chemistry and shed light on the potential role of silicate minerals in the synthesis of complex organic molecules derived from HCN-derived compounds in astronomical environments.

## Supporting Information Available

Zenodo repository with all structures modeled in this work (DOI: 10.5281/zenodo.14276127), benchmark on different density functionals for computing activation barriers and reaction energies, theoretical background of the RRKM kinetic theory, slab models adopted in the work, scaling factor for the vibrational modes of an isolated IAN molecule, and IR spectral features of adsorbed products (PDF)
