# Supplementary material for: Exploring Forsterite Surface Catalysis in HCN Polymerization: Computational Insights for Astrobiology and Prebiotic Chemistry
Source: ACS Earth Space Chem. 2025 Jan 17;9(2):303–13. doi: 10.1021/acsearthspacechem.4c00282 (PMC11849040; doi:10.1021/acsearthspacechem.4c00282)
Supplement: Supplementary file 1 — sp4c00282_si_001.pdf [file sp4c00282_si_001.pdf]

Exploring Forsterite Surface Catalysis in HCN Polymerization: Computational Insights for Astrobiology and Prebiotic Chemistry

Supporting Information

Niccolò Bancone [†,‡], Stefano Pantaleone [‡], Piero Ugliengo [‡], Albert Rimola [*,†] and Marta Corno [*,‡]

†*Departament de Química, Universitat Autònoma de Barcelona, Bellaterra, 08193, Catalonia, Spain*

‡*Dipartimento di Chimica and Nanostructured Interfaces and Surfaces (NIS) Centre, Università degli Studi di Torino, via P. Giuria 7, 10125, Torino, Italy.*

E-mail: albert.rimola@uab.cat; marta.corno@unito.it

Phone: +34-935813723; +39-0116702439

Computational Details

Benchmark

Table S1: Activation barriers ($\Delta E^{\ddagger}$) and reaction energies (ΔE^{*}) for the 2HCN → IAN reaction on the $(Mg_2SiO_4)_3$ nanocluster, computed with different DFT functionals and DLPNO-CCSD(T)/aug-cc-pvtz. All the geometries were generated at the PBE-D*N level of theory, adopting the Ahlrichs VTZP basis set for H, C and N, and a Pople-like basis set for Mg, Si and O, which was used also for all single points performed with DFT. The DLPNO-CCSD(T) calculations were performed with the ORCA program, version 5.0. All the other calculations reported here were performed with CRYSTAL17. For each value, the absolute percentage deviation with respect to the DLPNO-CCSD(T) (|Δ%|) is reported.

Method	$\Delta E^{\ddagger}$	(|Δ%|)	ΔE^{*}	(|Δ%|)
DLPNO-CCSD(T)	37.4		32.1	
B3LYP-D3	25.7	(31.2%)	18.7	(41.8%)
B97-D3	16.9	(54.9%)	11.2	(65.2%)
BHLYP-D3	38.8	(3.8%)	26.9	(16.1%)
PBE0-D3	20.0	(46.4%)	0.6	(98.2%)
PW6B95-D3	21.8	(41.7%)	2.7	(91.5%)
PWP1	20.9	(44.1%)	2.6	(92.0%)
mPW1PW	21.6	(42.2%)	4.2	(86.8%)

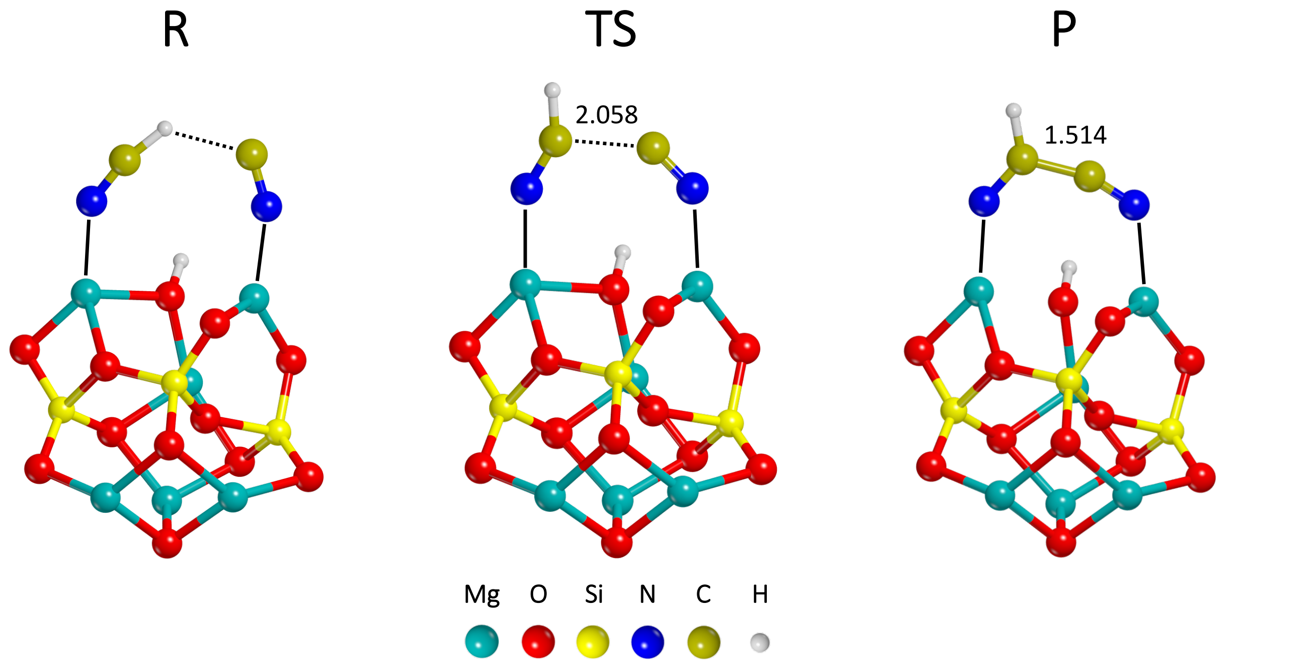

Figure S1: Models adopted for the benchmark of different DFT methods described in Table S1. Two HCN molecules, one deprotonated, are adsorbed on a small forsterite cluster (three Mg_2SiO_4 units) and react to create a deprotonated IAN. R, TS and P stand for reactant, transition state and product, respectively. The C–C distances in TS and P are reported in Å.

Surface adsorption sites

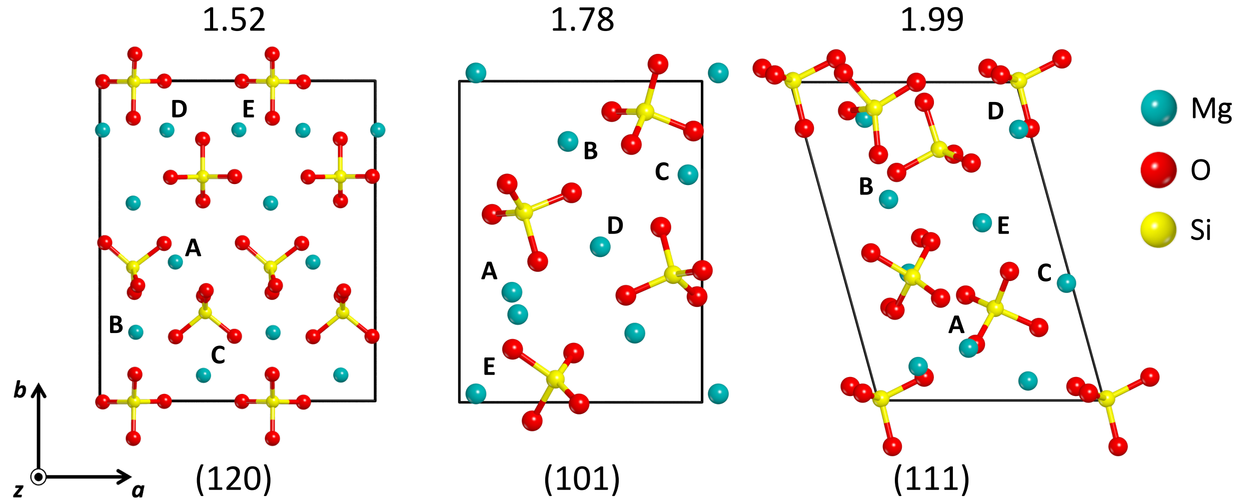

Figure S2: Top views of the slab models adopted in this work. For the sake of clarity, only the first layers of the models are depicted. On the top of each surface, the relative surface energy in J m^{-2} is reported. Mg^{2+} cations available to adsorption are labeled according to the conventions proposed in ref. 1,2, A being the most and E being the least exposed ones.

RRKM theoretical background

Here the theoretical background of the unimolecular kinetic code based on the RRKM theory[3–6] is briefly described.

Being E_0 the energetic barrier of a generic reaction step, the associated microcanonical rate constant $k(E)$ at a given total energy $E > E_0$ is given by

$$k(E) = \frac{N^{\neq}(E - E_0)}{h\rho(E)} = \frac{\int_{-E_0}^{E-E_0} T(\xi^{\neq})\rho^{\neq}(E - E_0 - \xi^{\neq})d\xi^{\neq}}{h\rho(E)} \tag{1}$$

where $N^{\neq}(E-E_0)$ is the sum of states for the TS, $\rho(E)$ is the density of states of the reactants and h the Planck constant. $N^{\neq}(E - E_0)$ can be re-written as an integral of the density of states of the TS over the translational energy of the reaction coordinate $\xi^{\neq}$. $T(\xi^{\neq})$ represents the transmission probability through the potential energy barrier and it is introduced into the integral in order to take into account tunneling effect. It can be computed by mean of an asymmetric Eckart potential:[7,8]

$$T(\xi^{\neq}) = \frac{cosh(a + b) - cosh(a - b)}{cosh(a + b) + cosh(\sqrt{4\alpha_1\alpha_2 - \pi^2})} \tag{2}$$

with $a = 2(\alpha_1\zeta)^{1/2}(\alpha_1^{-1/2} + \alpha_2^{-1/2})^{-1}, b = 2[(\zeta - 1)\alpha_1 + \alpha_2]^{1/2}(\alpha_1^{-1/2} + \alpha_2^{-1/2})^{-1}, \alpha_1 = 2\pi V_1/h\nu^{\neq}, \alpha_2 = 2\pi V_2/h\nu^{\neq}, \zeta = E/V_1$, where V_1 is the barrier from the TS to the reactants, V_2 the barrier from the TS to the products and $\nu^{\neq}$ the absolute value of the imaginary frequency associated with the TS.

If the energy levels of the system are populated according to a Boltzmann distribution $P(E)$, then the microcanonical $k(E)$ can be finally converted into the canonical $k(T)$

$$k(T) = \int_{E_0}^{\infty} k(E) \times P(E)d(E) = \int_{E_0}^{\infty} k(E) \times \frac{g(E)exp(-E/k_bT)}{Q(T)}dE \tag{3}$$

Results

Surface energies and nanoparticle shape

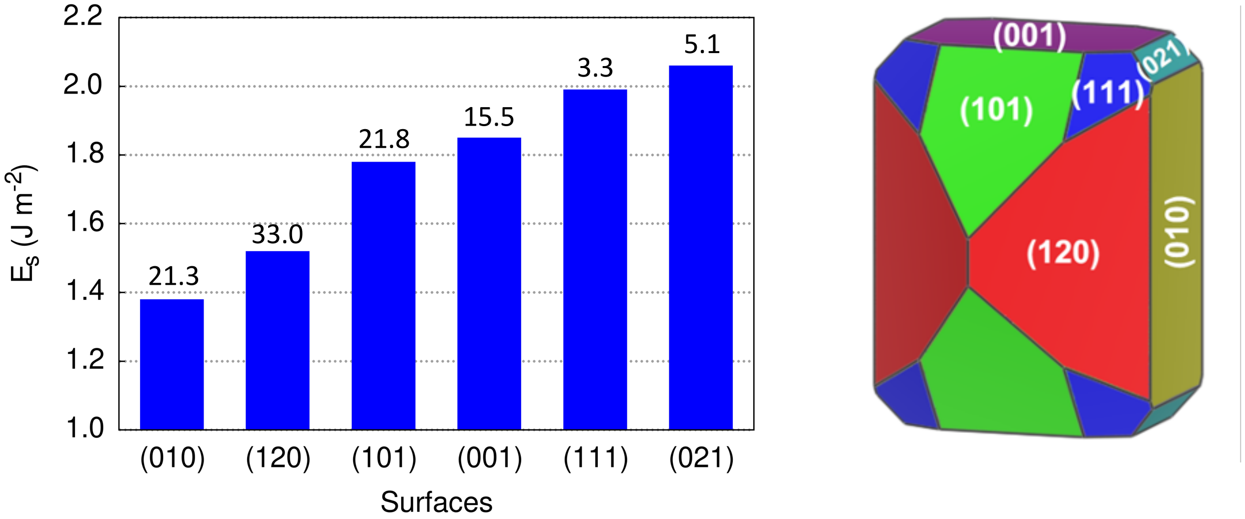

Figure S3: Left: surface energy of the six slab models calculated in our previous work at the PBE-D*N level. The respective morphological relevance index (MRI) values are reported on top of each bar. Right: Wulff's construction of a forsterite nano-crystal at 0 K.[2]

Spectral features of the products

The spectroscopic features of the products were evaluated after correcting the computed IR stretching frequencies of each mode for the corresponding scaling factor (s), obtained as the ratio between the experimental[9] and the computed frequency of an isolated iminoacetonitrile molecule:

$$s_i = \frac{\bar{\nu}_i^{exp}}{\bar{\nu}_i^{theo}} \tag{4}$$

where i denotes the i-esimal mode (see Table S2).

In this section we provide a brief description of the computed vibrational modes of the products of the reactions, for the benefit of future works on this topic. The computed frequencies, corrected for their corresponding scaling factor s_i (see Table S2), of the adsorbed

Table S2: Scaling factors s_i for the vibrational modes of the IAN molecule obtained as the ratio between the computed frequencies in the gas phase (Comp.) and the experimental ones (Exp.).

Mode	Comp.	Exp.[9]	s_i
C-H oop	821	815	0.9927
C-C str.	902	904	1.0021
C=N tor.	1101	1095	0.9949
CNH bend	1202	1218	1.0134
C-H bend	1377	1388	1.0076
C=N str.	1607	1599	0.9951
CN str.	2243	2239	0.9980
C-H str.	3050	2943	0.9651
N-H str.	3339	3306	0.9901

IAN^- (IAN for the (120) BC case) obtained in the 6 reactions are reported in Table S3, while the corresponding structures are in Fig. S 4. The computed frequencies of the adsorbed products are compared with the experimental ones for a IAN molecule in the gas phase[9] in order to evaluate the perturbations of the bonds of the molecule due to the anionic nature of the species and/or their interaction with the surfaces.

Focusing on the N–H stretching frequencies, the only case including this feature is (120) BC, as all other ionic IAN^- do not posses a N–H group. It results that the N–H bond in (120) BC is weakened by a H-bond donation to an exposed silicate of the surface (see Fig. S 4), determining an elongation of the N–H bond from 1.033, in the gas phase, to 1.057 Å, on the surface.

For what concerns the C–H stretching, this feature is comparable between the gas phase and the IAN in (120) BC, with a slight blue-shift ($\Delta\bar{\nu} = +12$ cm^{-1}) of the adsorbed molecule with respect to the gaseous one. On the opposite, all the C–H frequencies of the adsorbed IAN^- result to be red-shifted with respect to the gaseous IAN, with variations between -50 and -150 cm^{-1}. Accordingly, a slight shortening of the bond is observed in (120) BC (-0.001 Å with respect to the gaseous molecule) while all the ionic cases show an elongation of the

bond between +0.002 and +0.009 Å.

All the C≡N stretching frequencies undergo bathocromic shifts upon interacting with the surface. The effect is relatively light in (120) BC ($\Delta\bar{\nu}$ = -12 cm^{-1}) and more pronounced in the anionic cases ($\Delta\bar{\nu}$ = -53 – -202 cm^{-1}). In gaseous IAN, together with the stable (120) BC and (101) BC cases, the C≡N vibrates above 2200 cm^{-1}. The C≡N stretching frequency of gaseous HCN, instead, is 2097 cm^{-1}. Indeed, the occurrence of IR signals above 2200 cm^{-1} can be an indicator of reactivity in a pure HCN-forsterite system as, for the bare adsorption, that zone of the spectrum is not populated.[10,11]

The C=N stretching frequencies is red-shifted in (120) BC and blue-shifted in all other cases, with the only exception of (111) BD ($\bar{\nu}$ = 707 cm^{-1}). The latter is explainable in term of the hybridization of the C atom adjacent to N: while in all cases the nucleophilic addition of CN^- to HCN determines a decreasing of the bond order of C≡N to C=N, in (111) BD the C atom is also bound to a superficial O, determining a further decreasing to a single C–N bond.

Regarding the C–H and CNH bending and the C=N torsional modes, the last two are only present in the gaseous IAN and (120) BC, due to the presence of one more H atom in IAN with respect to IAN^-. The bending and torsional modes in (120) BC are all blue-shifted with respect to the gaseous IAN, due to the interaction with the surface which determines a more constrained structure of the adsorbate. Unlike (120) BC, in all other cases the C–H bending frequency of adsorbed IAN^- is red-shifted with respect to the neutral IAN.

The C–C bond is weakened with respect to the gas phase in all cases, with a smaller effect in (120) BC ($\Delta\bar{\nu}$ = -8 cm^{-1}) than in the other cases ($\Delta\bar{\nu}$ = -19 – -181 cm^{-1}). Lastly, the out-of-plane (oop) bending mode of the C–H group vibrates at a slightly lower frequency in (120) BC with respect to the gas ($\Delta\bar{\nu}$ = -7 cm^{-1}) while in the other cases it results blue-shifted ($\Delta\bar{\nu}$ = +18 – +69 cm^{-1}). This mode is not present in case (111) BD because of the C–O bond with the surface.

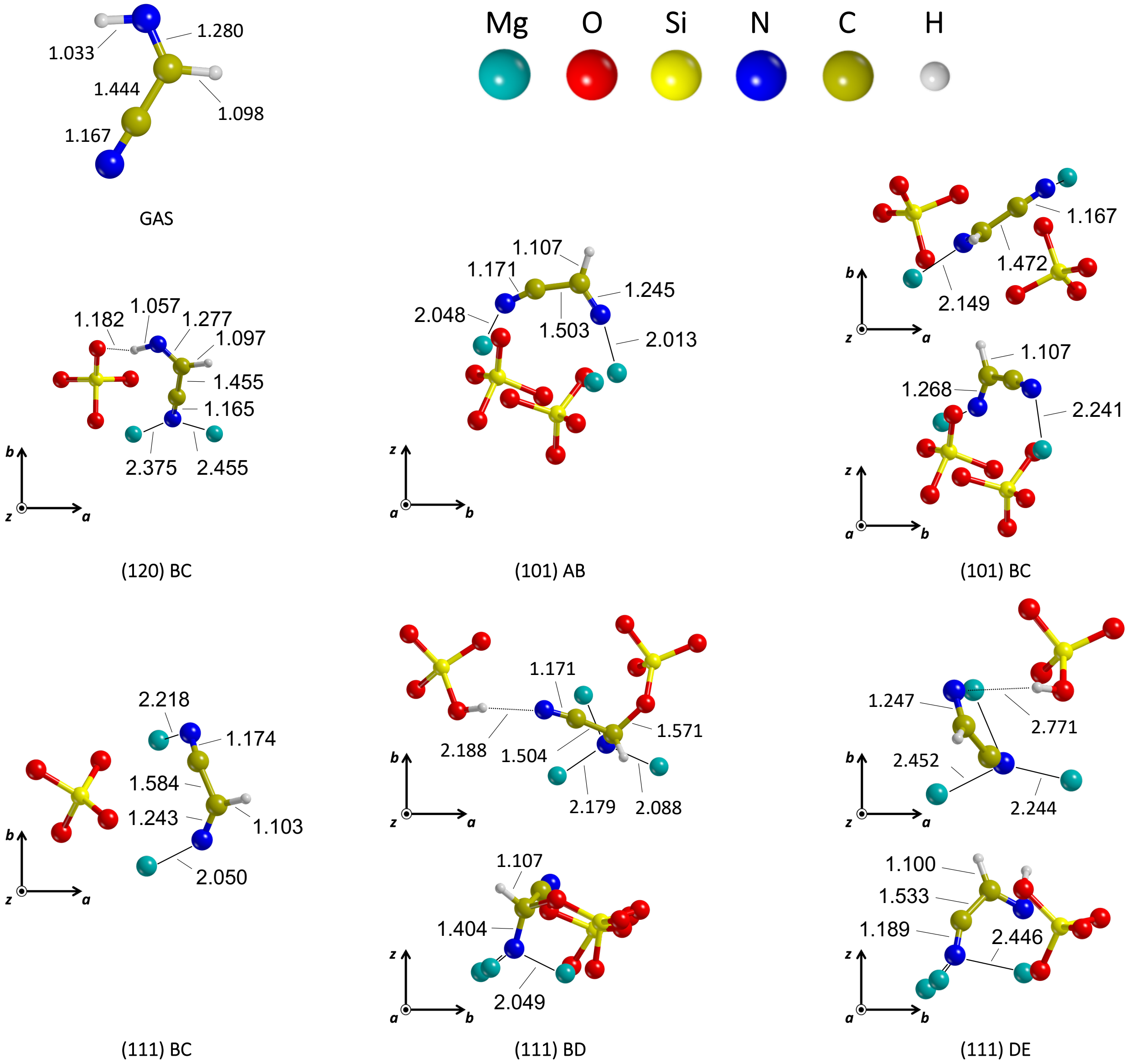

Figure S 4: Graphical representation of the adsorbed products (neutral IAN for GAS and (120) BC, ionic IAN- for the rest of cases), focusing on the adsorbate region. For the sake of clearness, both top and side views of cases (101) BC, (111) BD and (111) DE are illustrated. All distances are in Å.

Table S3: Computed IR frequencies of six cases of adsorbed IAN molecules on forsterite surfaces. The second column reports the experimental vibrational frequencies of a neutral IAN molecule in the gas phase. All frequencies are in cm^{-1}.

Mode	GAS[9]	(120) BC	(101) AB	(101) BC	(111) BC	(111) BD	(111) DE
N-H str.	3306	2912					
C-H str.	2943	2955	2806	2819	2856	2805	2895
C≡N str.	2239	2227	2179	2219	2123	2186	2037
C=N str.	1599	1577	1697	1605	1675	707	1636
C-H bend	1388	1400	1327	1351	1341	1280,1303	1277
CNH bend	1218	1309					
C=N tor.	1095	1171					
C-C str.	904	896	823,827	885	723	856,863,869	787
C-H oop	815	808	864	884	852		833

References

(1) Zamirri, L.; Corno, M.; Rimola, A.; Ugliengo, P. Forsterite surfaces as models of interstellar core dust grains: computational study of carbon monoxide adsorption. *ACS Earth Space Chem.* **2017**, *1*, 384–398.

(2) Bancone, N.; Pantaleone, S.; Ugliengo, P.; Rimola, A.; Corno, M. Adsorption of HCN on cosmic silicates: a periodic quantum mechanical study. *Phys. Chem. Chem. Phys.* **2023**, *25*, 26797–26812.

(3) Marcus, R. A.; Rice, O. The Kinetics of the Recombination of Methyl Radicals and Iodine Atoms. *J. Phys. Chem.* **1951**, *55*, 894–908.

(4) Marcus, R. A. Unimolecular dissociations and free radical recombination reactions. *J. Chem. Phys.* **1952**, *20*, 359–364.

(5) Rosenstock, H. M.; Wallenstein, M.; Wahrhaftig, A.; Eyring, H. Absolute rate theory for isolated systems and the mass spectra of polyatomic molecules. *Proc. Natl. Acad. Sci. U. S. A.* **1952**, *38*, 667–678.

(6) Baer, T.; Hase, W. L. *Unimolecular reaction dynamics: theory and experiments*; Oxford university press, 1996; Vol. 31.

(7) Eckart, C. The penetration of a potential barrier by electrons. *Phys. Rev.* **1930**, *35*, 1303.

(8) Johnston, H. S.; Heicklen, J. Tunnelling corrections for unsymmetrical Eckart potential energy barriers. *J. Phys. Chem.* **1962**, *66*, 532–533.

(9) Osman, O. I. Experimental and Theoretical Investigation of the Pyrolysis Products of Iminodiacetonitrile,$(N{\equiv}CCH_2)_2NH$. *J. Phys. Chem. A* **2014**, *118*, 10934–10943.

(10) Santalucia, R.; Pazzi, M.; Bonino, F.; Signorile, M.; Scarano, D.; Ugliengo, P.; Spoto, G.; Mino, L. From gaseous HCN to nucleobases at the cosmic silicate dust surface: An experimental insight into the onset of prebiotic chemistry in space. *Phys. Chem. Chem. Phys.* **2022**, *24*, 7224–7230.

(11) Bancone, N.; Santalucia, R.; Pantaleone, S.; Ugliengo, P.; Mino, L.; Rimola, A.; Corno, M. Unraveling the Interface Chemistry between HCN and Cosmic Silicates by the Interplay of Infrared Spectroscopy and Quantum Chemical Modeling. *J. Phys. Chem. C* **2024**, *128*, 15171–15178.
